# Eye-Closure Enhances Creative Performance on Divergent and Convergent Creativity Tasks

**DOI:** 10.3389/fpsyg.2018.01315

**Published:** 2018-07-31

**Authors:** Simone M. Ritter, Jens Abbing, Hein T. van Schie

**Affiliations:** Department of Behaviour Change and Well-Being, Behavioural Science Institute, Radboud University, Nijmegen, Netherlands

**Keywords:** creativity, divergent thinking, convergent thinking, eye-closure, alpha-power, working memory

## Abstract

In today’s world of rapid changes and increasing complexity, understanding and enhancing creativity is of critical importance. Studies investigating EEG correlates of creativity linked power in the alpha frequency band to creativity, and alpha-power has been interpreted as reflecting attention on internal mental representations and inhibition of external sensory input. Thus far, however, there is no direct evidence for the idea that internally directed attention facilitates creativity. The aim of the current study was to experimentally investigate the relationship between eye-closure—a simple and effective means to stimulate internally directed attention—and creativity. Moreover, to test whether the potential beneficial effect of eye-closure is specific for creativity, or whether it improves general cognitive functioning, the current study tested the effect of eye-closure on creativity and on working memory (WM). Participants completed four tasks to measure divergent and convergent creativity (Adapted Alternative Uses (AAU) Test, Remote Associates Test (RAT), Sentence Construction Test, and Word Construction Test), and one task to measure WM (Digit Span Test). For each task, participants had to perform two versions, one version with eyes open and one version with eyes closed. Eye-closure facilitated creative performance on the classical divergent and convergent creativity tasks (AAU Test and RAT). No effect of eye-closure was observed on the WM task. These findings provide a novel and easily applicable means to enhance divergent and convergent creativity through eye-closure.

## Introduction

Creative thinking is one of the most important cognitive skills in our fast-changing world (Ananiadou et al., 2009)—it allows us to remain flexible, and provides us with the capacity to deal with the opportunities and challenges that are part of our dynamic environment (Ritter and Mostert, 2017). In recent years, increasing insights have been gained into how creative ideas arise in the brain. In particular, creativity is found to be strongly associated with alpha oscillations in frontal and posterior parietal brain regions (see review in Fink and Benedek, 2014). Alpha oscillations are hypothesized to protect internal mental processes supporting creative thought from distracting external sensory information that may interfere with the creative process (Cooper et al., 2003). The current study tested the hypothesis that excluding external information through eye-closure will benefit creativity.

An increase in alpha power during creative ideation is among the most consistent findings in neuroscientific research on creativity (for a review, see Fink and Benedek, 2014). Already in the 1970s Martindale and Hines (1975) found that high creative individuals produced more alpha power during creativity tasks. Recent studies have confirmed and extended these initial findings. Alpha power during creative thinking has been found (i) to vary as a function of individual differences in creativity (Jaušovec, 2000; Fink and Neubauer, 2006; Fink et al., 2009a,b), (ii) to increase following a creativity training program (Fink et al., 2006a), and (iii) to vary as a function of the originality of creative ideas (Fink and Neubauer, 2006; Grabner et al., 2006). A study by Jung-Beeman et al. (2004) found that prior to gaining creative insight, individuals show a burst of activity in the alpha frequency, and studies using transcranial alternating current stimulation (tACS) have found creativity performance to increase when frontal regions were stimulated at the alpha frequency of 10 Hz (Lustenberger et al., 2015; Grabner et al., 2017).

Since the discovery of alpha by Berger (1929) the functional nature of the alpha rhythm has been a topic of scientific inquiry and theorizing. Early explanations suggested that alpha oscillations reflect cortical idling (Adrian and Matthews, 1934; Pfurtscheller et al., 1996a), that is, a default state of cortical inactivation, as for instance found during eye-closure (Toscani et al., 2010) and movement inactivity (Pfurtscheller et al., 1996b). However, more recent empirical findings have indicated that alpha plays an important functional role in human cognition by providing an inhibitory mechanism for gating irrelevant and distracting information from interfering with task specific processing (see reviews in Klimesch et al., 2007; Jensen and Mazaheri, 2010). For instance, studies investigating working memory (WM) have found alpha power to increase during the retention of information (e.g., Jensen et al., 2002; Tuladhar et al., 2007), which is taken to reflect an active inhibition of processes in brain areas that are not involved in WM (Suffczynski et al., 2001; Jensen and Mazaheri, 2010). In accordance with these findings, Cooper et al. (2003) suggested that alpha power reflects the active inhibition of external sensory input when tasks require attention to be directed internally such as in the case of mental arithmetic, mental imagery, or WM. Fink and Benedek (2014) hypothesize that a similar principle may explain the increase in alpha power that accompanies creative thinking.

Although alpha has been consistently found to be activated in association with creative thinking (e.g., Fink and Benedek, 2014) and many studies have confirmed the role of alpha in the inhibition of external distracting stimuli (e.g., Jensen and Mazaheri, 2010), so far, no direct evidence has been reported for the hypothesis that creative thinking is facilitated when external stimuli are suppressed. Benedek et al. (2011, 2014) investigated if masking of external stimuli shortly after their presentation would contribute to creative performance, forcing participants to use an internal processing strategy. Opposite to what was expected, their manipulation caused creative performance to deteriorate in tasks that require a repeated sampling of visual information, that is, in the four-word sentence task and the anagram solution task (Benedek et al., 2011, 2014). However, also no effect of the masking manipulation was found in the performance of the Alternative Uses Test (AUT), which can be performed without visual resampling. The present study aimed to investigate the hypothesis that creativity is enhanced through the suppression of external stimuli via a different method. More specifically, in the present study we manipulated eye-closure as simple and direct means to suppress external stimuli. Furthermore, to prevent the suppression of task-relevant stimuli, we presented stimuli in the auditory rather than the visual modality.

Previous studies have established eye-closure as an effective method to aid memory retrieval (e.g., Perfect et al., 2008; Mastroberardino et al., 2012; Mastroberardino and Vredeveldt, 2014) and to improve performance on mathematical queries and general-knowledge questions (Glenberg et al., 1998), arguably because eye-closure effectively reduces interference from external stimulation. A similar mechanism is considered to be responsible for gaze aversion, that is, the phenomenon that people direct their gaze away from salient stimuli such as the eyes of a conversation partner, when they engage in a cognitively demanding task such as memory retrieval (Glenberg et al., 1998; Phelps et al., 2006; Doherty-Sneddon et al., 2012). Interestingly, Salvi et al. (2015) recently investigated eye-gazes during creative problem solving. Prior to successful creative problem solving— – as compared to analytic problem solving—participants displayed fewer fixations on the screen (where the task was presented) and blinked longer and more frequently. These findings indicate that people are naturally inclined to suppress externally distracting information by averting their gaze, or blinking. The current study aims to further enhance these findings by investigating if manipulation of eye-closure will facilitate creative performance.

Importantly, eye-closure does not only directly suppress the participants’ immediate external visual environment, but is also known to stimulate alpha-activity (Geller et al., 2014), which may also suppress task-irrelevant information from interfering with the creative mental process. A study by Toscani et al. (2010) found that eye-closure enhanced alpha power over occipital areas as compared to a condition in which the eyes were open, even though both conditions were measured in absolute darkness. Furthermore, in the same study it was found that the increase in alpha activity accompanying eye-closure suppressed the detection of visual stimuli delivered to the retina through the oral cavity using an endoscopic LED mouth piece. These findings confirm the inhibitory role of alpha in visual perception and indicate that eye-closure directly affects alpha amplitude. Hence, we hypothesize that eye-closure will facilitate creative thought, either directly by the suppression of external visual information, or indirectly by enhancing alpha power.

Creativity entails both divergent and convergent thinking (e.g., Finke et al., 1992; Runco and Basadur, 1993; Basadur, 1995; Lubart, 2001; Simonton, 2003; Reiter-Palmon and Illies, 2004; Sawyer, 2006; Sternberg, 2006; Ritter et al., 2012a). Divergent thinking is the capacity to generate multiple ideas or solutions, and divergent thinking tasks are the most widely used creativity tests (Cropley, 2000; Davis, 2003). Convergent thinking is the cognitive process of deriving the best answer to a problem or question (Guilford, 1967; Treffinger, 1995; Nickerson, 1999; Fasko, 2001). Given that creativity entails both divergent and convergent thinking, adapted auditory versions of the AUT and the Remote Associate Test (RAT) were included as standard tests of divergent and convergent creativity in the current study. In addition we included auditory versions of the divergent four-word sentence task and the convergent anagram solution task that were used by Benedek et al. (2011, 2014), as these tasks strongly depend on WM for representing and manipulating the consecutive letters of the stimulus words. Lastly, a standard Digit Span (DS) test was included to control for unspecific (e.g., cross-modal enhancement) effects of eye-closure on verbal WM, which could also contribute to the performance in the creativity tests. The tasks are described in more detail below.

The present study aims to enhance our scientific understanding of creativity and to provide important practical implications. During the last years, several creativity enhancement techniques have been developed (Ritter et al., 2012a,b; Ritter and Ferguson, 2017; for a review, see Scott et al., 2004)—many of them are cost and time intensive. Eye-closure, however, may function as an easily applicable means to enhance creative thinking that can be applied in various educational and organizational settings where creative thinking is needed.

## Materials and Methods

### Participants

A total of 38 participants (85% females) between the ages of 18 and 22 years old (*M* = 19.7 years) gave written informed consent to participate in the study. No *a priori* power analysis was calculated. As a stopping rule we decided to include as many participants as possible within the available time that the lab was reserved. The study was conducted according to the principles expressed in the Declarations of Helsinki and according to the guidelines of the institutional review board (Ethics Committee Faculty of Social Sciences, Radboud University, Nijmegen, Netherlands). Ethical approval was at the time of data collection not required by the Institution’s guidelines and national regulations, as the research was not of a medical nature, no minors or persons with disability were involved, and there were no potential risks to the participants. All the participants were German and recruited for voluntary participation via the online research participation system (Sona) of Radboud University or on campus. Participants earned course credit (1 point) for their participation. Six participants had to be excluded from the analysis [due to unrecorded answers as the result of a microphone failure (*n* = 4), falling asleep during the experiment (*n* = 1), or taking notes while performing the tasks (*n* = 1)], resulting in a total of 32 participants that were included in the analysis. *Post hoc* computation of achieved power in G^∗^Power (Faul et al., 2009) indicated that with 32 participants the study was slightly underpowered, with the power varying between 0.62 [Adapted Alternative Uses (AAU)] and 0.78 (RAT).

### Instruments

Five different tasks were used. To measure divergent thinking performance, an AAU test and a Sentence Construction (SC) test were administered. To measure convergent thinking, a RAT and a Word Construction (WC) were administered. The exact list of words and prompts used in the divergent and convergent thinking tasks are described in the **Supplementary Table S1**. To measure WM performance, a DS test was examined. All tasks were based on previous research and adapted to meet the requirements of the current experiment (e.g., materials in German and multiple items per task).

### Material Development

For each task, 90 items were created and examined in an online pre-test. Due to the large number of items, each task was split in two versions, each consisting of 45 items. The 45-item versions were each completed by 20 participants. Items that were successfully solved by 10% or more than 90% of participants were excluded. Furthermore, items with multiple solutions in the RAT and the WC test were excluded. For each task, 30 items were selected for the actual experiment. The items were selected based on their difficulty so they could be divided in two equally difficult versions of 15 items. One version was completed with eyes closed and the other with eyes open. The order was counterbalanced across participants. For each task, a fixed response window was determined based on the pre-test.

### Creativity Tasks

The AUT (Guilford, 1967) and the RAT (Mednick, 1962) were included, as they are the most frequently used divergent and convergent thinking tests, respectively. In addition to these classic tasks, a SC test and a WC test were administered. These latter tasks were developed by Benedek et al. (2011) as additional measures of divergent and convergent thinking, respectively. The four tasks are described in more detail below.

#### Adapted Alternative Uses Test (AAU)

This test represents an adapted version of the AUT by Guilford (1967). Consistently with the other tasks, in the current study it consists of multiple short measurements instead of one or two lengthy ones, as in the original AUT. In this version, participants heard the name of an object and were asked to name three new applications for this object. For example, the item could be an iron and participants could answer “I use it to heat up my sandwich,” “I melt vanilla ice to create a drink,” and “I use it as an improvised radiator.” Participants were given 20 s for their response, and one point was awarded for each correct answer, resulting in a maximum of three points per provided item. The final score was the sum of correct answers across the 15 items (0–45 points).

#### Remote Associates Test (RAT)

In this convergent thinking task, three stimulus words were presented verbally via the headphone, and participants had to name a fourth word that is related to all three words within 15 s. For example, participants were presented with the words “tree – yellow – sour.” The correct response to this would be “lemon,” as it is associated with all stimulus words. This test was created by Mednick (1962), but in line with suggestions by Worthen and Clark (1971), associations based on homonyms (words that have the same pronunciation but different meanings, e.g., shooting star and movie star) were not included. As English three-word combinations are rather difficult for non-native speakers (e.g., Estrada et al., 1994), in the current study, German word triads were used. The total score of this test was the number of correct answers (0–15 points).

#### Sentence Construction (SC)

In this test, participants heard four letters and had to construct a sentence in which each word starts with one of these letters in the presented order. For example, with the letters “PDAC,” one could form the sentence “Policemen dislike attending conferences.” This task is based on the divergent thinking task used by Benedek et al. (2011). Participants had 10 s to respond with a grammatically correct sentence and the number of correct sentences was used as the total score (0–15 points).

#### Word Construction (WC)

Participants heard four letters and had to find a word that consisted of these four letters. For example, the letters could be “L – O – C – D” and “cold” would be the correct solution. This task is derived from the study by Benedek et al. (2011). Participants had a response window of 10 s for their answer. The final score was the number of correct answers (0–15).

### Working Memory Task

#### Digit Span (DS)

In this task, participants had to repeat sequences of random numbers that were presented auditory. The first sequence had a length of four numbers. Subsequent sequences were increased in length by one until the final sequence that consisted of nine numbers. Hence, a total of six sequences were presented consisting of four, five, six, seven, eight, and nine numbers that had to be repeated by the participants following auditory presentation. Participants were given 8 s to respond to the four characters item, plus 1 s for each additional character. The final score was the number of correct items (0–6). Auditory DS tasks have been used to assess phonological WM and are highly reliable (Drewnowski and Murdock, 1980).

### Original Alternative Uses Test (AUT)

To validate the adapted creativity tests in the current experiment, participants also completed a regular AUT, which has been widely recognized as a reliable and valid measurement of creative potential (Runco and Acar, 2012). This task was not presented auditory but was completed with pen and paper on an A4 sheet, with short written instructions. The participants were given 3 min to list their ideas. By coding the listed ideas, the participants’ cognitive flexibility and originality was assessed. Cognitive flexibility reflects the number of distinct idea categories used. Each idea was assigned to a category from a pre-defined list of categories, and the total number of distinct idea categories was then calculated per participant. Originality was measured by evaluating the uniqueness of the answers in the sample. An answer that was given by no other participant was rated with three points, an answer given by <10% of the participants with two points, and all other answers with one point (Benedek et al., 2013). In line with suggestions by Benedek et al. (2013), participants were asked to select their three most creative answers, and the average rating of these items was used as the originality rating. This prevents the inclusion of additional, less original ideas from negatively affecting the rating. Otherwise, someone with five unique ideas and five common ideas would receive a lower rating than someone who just had five unique ideas.

### Procedure

Participants were welcomed at the lab entrance and accompanied to an individual testing-room where they were seated in front of a computer. First, short instructions for the upcoming tasks were presented with two example items and solutions per task. Thereafter, participants had to complete two practice items (an easy and a difficult item) per task in the presence of the researcher. If necessary, the researcher provided feedback to ensure that participants understood the tasks correctly. Subsequently, the light was slightly dimmed so that eye-closure would be effective in suppressing external light sources, and the researcher left the room. For each item, participants heard an auditory stimulus through headphones that had been recorded (e.g., the words “tree-yellow-sour” for the RAT). After exposure to the auditory stimulus, participants heard a sound, and they could start to articulate in their response in the microphone. The end of the response-time was marked with a sound. Within the response-timeframe, participants were allowed to correct their answer. For each task, participants were presented with two auditory blocks of 15 items per block. During one block participants were instructed to close their eyes, whereas during the other block they were instructed to keep their eyes open. Eye-closure was counterbalanced, so half of the participants closed their eyes during the first block, and the other half of the participants during the second block. The order of items within the set was randomized per participant. The order of eyes-open and eyes-closed was consistent across the five tasks. After having completed the five auditory tasks, participants completed the written Alternative Uses control test. All participants completed the tasks in the same order (RAT, SC, AAU, WC, DS, and AUT). In total, the experiment lasted approximately 1 h and 10 min.

### Statistical Analysis

Due to the difference in absolute performance between the tests, the outcome variables were centered around the mean for each test, so that all measures had an average of zero. For the AAU test, the final score was divided by three to match the scale of the other tasks. A principal component analysis was conducted to investigate whether creativity and WM tasks indeed measured two distinct abilities. To test the effect of eye-closure on creativity and WM performance, a two-factor repeated-measures ANOVA was conducted with *Score* (the performance on each task) as the continuous outcome variable and the factors *Eyes* (open or closed) and *Test* (the five tasks).

## Results

### Descriptive Statistics

An overview of the measures used in this study are provided in **Table 1**.

**Table 1:** Descriptive statistics of the creativity tasks and working memory task.

Measure	Theoretical	Actual	*M* (SD)
	range	range	
Adapted Alternative Uses (AAU)	0–45	16.50–44.50	32.84 (7.42)
Remote Associates Test (RAT)	0–15	4–12.50	7.50 (2.06)
Sentence Construction (SC)	0–15	2.50–13.00	7.33 (2.74)
Word Construction (WC)	0–15	3.50–13.50	8.63 (2.57)
Digit Span (DS)	0–6	2–5	3.20 (0.66)
AUT – Flexibility (AUT-F)	0–∞	3–9	5.48 (1.50)
AUT – Originality (AUT-O)	1–3	1.33–2.67	2.00 (0.38)


### Correlations

The correlations between the different creativity tasks and the WM task are displayed in **Table 2**. The four creativity measures were all positively correlated (0.030 < *p* < 0.001), except for the relation between RAT and WC (*p* = 0.233). The DS, was correlated to all auditory tasks that require participants to hold multiple items in their WM, namely the RAT (*p* = 0.030), the SC test (*p* = 0.025), and the WC test (*p <* 0.001).

**Table 2:** Correlations between the auditory tasks.

Measure	AAU	RAT	SC	WC	DS
AAU	/				
RAT	0.558^∗∗^	/			
SC	0.619^∗∗^	0.559^∗∗^	/		
WC	0.347^∗^	0.217	0.617^∗∗^	/	
DS	0.230	0.384^∗^	0.395^∗^	0.582^∗∗^	/


*^∗^*p* < 0.05, ^∗∗^*p* < 0.01.*

In **Table 3**, the correlations between the five auditory tasks (i.e., the four creativity measures and the WM test) and the original AUT are presented. The validity of the AAU task was confirmed by the correlation with both the cognitive flexibility (*p* = 0.001) and the originality (*p* = 0.001) dimension of the original AUT. Furthermore, the flexibility dimension of the AUT was positively correlated with the RAT (*p* = 0.047), and the originality dimension of the AUT was correlated to the SC test (*p* = 0.002) and the WC test (*p* = 0.042).

**Table 3:** Correlations between the auditory tasks and the original Alternative Uses Task.

	AUT flexibility	AUT originality	AAU	RAT	SC	WC	DS
AUT flexibility	/	0.500^∗∗^	0.563^∗∗^	0.354^∗^	0.330	0.185	0.198
AUT originality	0.500^∗∗^	/	0.542^∗∗^	0.532^∗∗^	0.542^∗∗^	0.362^∗^	0.147


*^∗^*p* < 0.05, ^∗∗^*p* < 0.01.*

### Principal Component Analysis

To investigate whether the creativity tests and the WM test measure two distinct constructs, a principal component analysis was conducted on the four creativity measures, the WM measure, and the two outcome variables of the original AUT (see **Table 4**). The sampling adequacy was assessed with the Kaiser-Meyer-Olkin measure, *KMO* = 0.721 (“good” according to Field, 2009). Bartlett’s test for sphericity indicated that correlations between items were sufficiently large to justify a principal component analysis, χ^2^(21) = 84.50, *p* < 0.001. Two factors had eigenvalues above Kaiser’s criterion of 1 (3.47 and 1.20), and in combination they explained 66.8% of the variance. After rotation, the factor loadings displayed in **Table 4** were obtained. The combination of items that load highly on each factor suggest that factor 1 can be interpreted as creativity and factor 2 as WM. The AAU, RAT, AUT-flexibility, and AUT-originality have factor loadings on the creativity (CR) component and do not load on the WM component. The DS measure loads on the WM component but not on the CR component. Surprisingly, the WC task loads strongly on the WM component and not on the CR component, which suggests that this task is not representative as a measure of creativity but rather as a measure of WM. Furthermore, the SC task loads on both the creativity and the WM components, which suggests that this task does not provide a clear-cut measure of creativity but is reflecting both creativity and WM.

**Table 4:** Summary of principal component analysis on the five auditory tasks and the Alternative Uses Test.

Test	Factor	
	
	CR	WM
AAU	0.848	0.058
RAT	**0.538**	0.247
SC	**0.523**	**0.514**
WC	0.053	**0.847**
DS	-0.090	**0.886**
AUT – flexibility	**0.843**	-0.178
AUT – originality	**0.776**	-0.010


### Main Analysis

A two-factor repeated measures design was used to compare the effect of eye closure on performance for the four creativity tasks and the WM task. Mauchley’s test indicated a violation of sphericity for the main-effect of Task, χ^2^(2) = 110.41, *p* < 0.001, and the interaction between Eyes and Task, χ^2^(2) = 36.26, *p* < 0.001. Degrees of freedom were therefore corrected using Greenhouse Geisser estimates of sphericity (ε = 0.361 for the main-effect of Test and ε = 0.606 for the interaction). Tests of within-subjects effects indicated a main effect of Task, *F*(1.443,44.720) = 419.34, *p* < 0.001, $\eta_{p}^{2}$ = 0.931, reflecting the different ranges of the five tests. The main-effect of Eyes was found to approach significance, *F*(1,31) = 3.97, *p* = 0.055, $\eta_{p}^{2}$ = 0.114, indicating that across all tests, performance was slightly, but not significantly, better when participants closed their eyes. Importantly, the interaction between Eyes and Task was significant, *F*(2.425,75.186) = 4.20, *p* = 0.013, $\eta_{p}^{2}$ = 0.119, indicating that the effect of eye closure on performance differed between the five measures.

**FIGURE 1:**
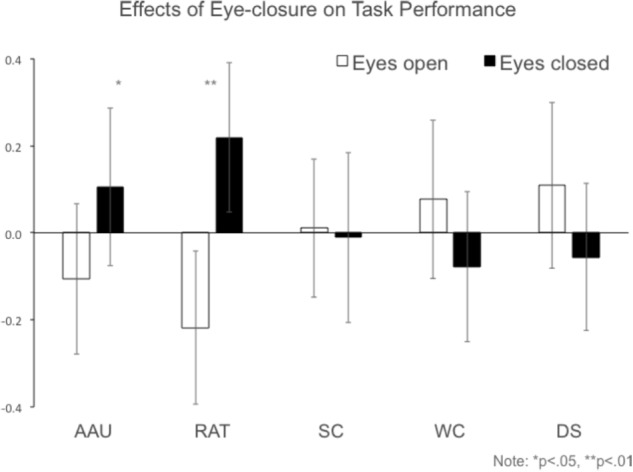
Effects of eye-closure on task performance in the creativity tests and the working memory measure. Performance values are normalized as *z*-scores for presentation. Error bars reflect the standard error of the mean. *p*-Values reflect the outcome of paired *t*-tests between eyes-open and eyes-closed for AAU, RAT, and SC, and the Wilcoxon signed-rank test for the WC and the DS.

**FIGURE 2:**
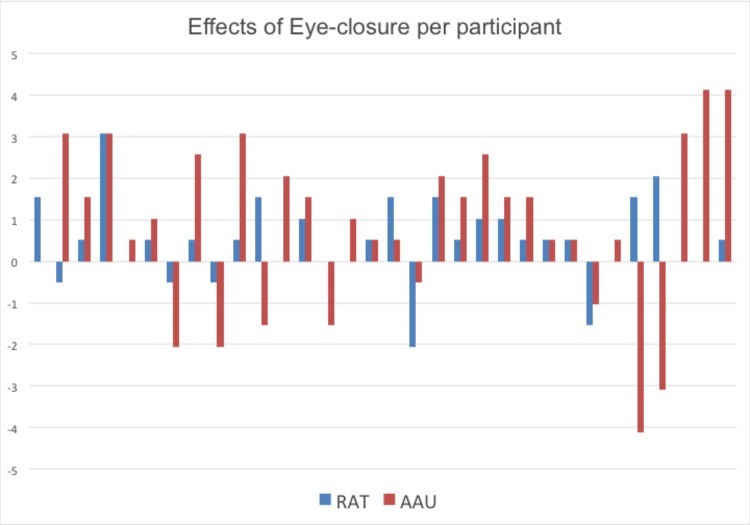
Effects of eye-closure per participant. Stacked plots of normalized individual difference scores (eyes closed – eyes open/standard deviation) in performance on the Remote Associate Test (RAT; in *blue*) and the Adapted Alternative Uses test (AAU, in *orange*). Each stacked bar presents the performance benefit (positive score) or performance disadvantage (negative score) for completing the tasks with eyes closed relative to eyes open.

Prior to investigating effects of eye-closure per task, difference scores between eyes open and eyes closed were calculated per task and checked for outliers and normality. Outlier detection was done using the boxplot function in SPSS to detect values larger or smaller than three times the interquartile range from the median. No outliers were detected. Normality was checked with the Shapiro–Wilk function. The distributions of difference scores did not deviate from normality for AAU, RAT, and SC. However, difference scores for the WC and DS were found to deviate from normality, *p* = 0.032 and *p* < 0.001, respectively. Paired samples *t*-test revealed that there was a positive effect of eye-closure on AAU task performance, *t*(31) = 2.328, *p* = 0.027, $\eta_{p}^{2}$ = 0.149, and on RAT performance, *t*(31) = 2.821, *p* = 0.008, $\eta_{p}^{2}$ = 0.204 (see **Figure 1**). In **Figure 2**, a graphical overview of the effects of eye-closure per participant on AAU and RAT performance is provided. No significant effect of eye-closure was found for the SC task, *t*(31) = 0.188, *p* = 0.852, $\eta_{p}^{2}$ = 0.001. Non-parametric tests indicated that there was no effect of eye-closure on WC test performance, *p* = 0.502, *r* = 0.083, and DS test performance, *p* = 0.132, *r* = 0.0189.

## Discussion

### General Discussion

One of the most consistent findings in neuroscientific research on creativity is the observed increase in alpha power during creative ideation (for a review, see Fink and Benedek, 2014). Alpha oscillations are hypothesized to protect internal mental processes supporting creative thought from distracting external sensory information that may interfere with the creative process (Cooper et al., 2003). Increased alpha power has been consistently found in association with creative thinking (e.g., Fink and Benedek, 2014), and many studies have confirmed the role of alpha in the inhibition of external distracting stimuli (e.g., Jensen and Mazaheri, 2010). However, to the best of our knowledge, no direct evidence has been reported for the hypothesis that creative thinking is facilitated by eye-closure—a simple and direct means to suppress external stimuli. The present study aimed at investigating the effect of eye-closure on divergent and convergent creativity. Moreover, to test whether the potential beneficial effect of eye-closure is specific for creativity, or whether it improves general cognitive functioning, the current study tested the effect of eye-closure on creativity and on WM. Participants completed four creativity tasks—adapted auditory versions of the AUT and the RAT were included as standard tests of divergent and convergent creativity, respectively; and in addition, two more recently developed creativity tasks were used, the auditory versions of the divergent four-word sentence task and the convergent anagram solution task. A standard DS test was included to control for unspecific (e.g., cross-modal enhancement) effects of eye-closure on verbal WM, which could also contribute to the performance in the creativity tests. For each of the five tasks, participants had to perform two versions, one version with eyes open and one version with eyes closed. Eye-closure facilitated creative performance on the classical measures of divergent and convergent creativity, that is, on the adapted version of the AUT and on the RAT. Importantly, no effect of eye-closure was observed for the WM tasks. The current findings provide first evidence for the effect of eye-closure on creativity and, moreover, suggest that the beneficial effect of eye-closure is specific to creativity and does not affect tasks that depend primarily on WM capacity. Whereas earlier research has mainly found correlational evidence—for example, it has been shown that alpha power varies as a function of individual differences in creativity (Jaušovec, 2000; Fink and Neubauer, 2006; Fink et al., 2009a,b) and as a function of the originality of creative ideas (Fink and Neubauer, 2006; Grabner et al., 2006)—the current study is the first one that provides *direct* evidence for the idea that the suppression of external information and the related increase in alpha enhances creative thinking.

### Limitations and Suggestions for Future Research

While the current study provides evidence that eye-closure is beneficial for performance on classical measures of divergent and convergent creativity, there are some limitations of the study that should be addressed in future research. First, the beneficial effect of eye-closure on creativity was observed for the two classical measures of creativity, the AUT and the RAT, but no effect was observed for the additional measures of divergent and convergent thinking (i.e., the SC test and the WC test). The principal component analysis conducted in the current study suggests that the WC test is a WM task, and that the SC test relies on both WM and creativity. Given that SC and WC substantially rely on WM, they do not seem ideal candidates for pure additional or alternative measures of divergent and convergent creativity. For future research it may be interesting to test the effect of eye-closure on additional divergent and convergent creativity tasks. Second, we cannot rule out the possibility that participants could have guessed that their creative performance should be better with eyes closed. However, as for the tasks administered in the current study creative performance does no rely on a participant’s fluency (the number of responses provided) it is unlikely that increasing effort leads to better performance. Moreover, when looking at the theoretical range and the actual range of participants’ task performance (**Table 1**), no extreme scores can be observed, ruling out the possibility that there was a lock of task performance in the eyes open condition. Third, the current sample consisted of students and had a limited age range of 18–22 years, and a relatively high proportion of females and western participants, which could limit the ecological validity of this study. On the other hand, findings of a meta-analysis by Scott et al. (2004) suggest that means to enhance creativity have greater effects on men than on women, and are more effective in organizational than academic settings. Considering that this study relied on a population and setting for which the *a priori* chance of finding a stimulation effect of eye-closure on creativity was not high, the ecological validity and generalizability of the current findings may be enhanced. However, it is still unknown what impact eye-closure would have on eastern participants’ creativity and on other age groups, for example, school-aged children and elderly people. Third, despite finding a positive effect of eye-closure, some participants performed better with open eyes. Due to the focus on within-subjects effects, the sample size of the present study was not adequate for a detailed investigation of individual differences. Additional studies should consider individual differences such as personality traits (e.g., openness to experience and extraversion) that could be responsible for diverging effects of eye-closure. Finally, future research could focus on enhancing our understanding of the underlying mechanism of eye-closure, internally directed attention, and creativity by using EEG measures or by focusing on fMRI correlates—one could, for example, investigate whether brain regions related to unconscious, automatic processes and/or intentional, imaginative processes are active during creativity tasks with eyes closed versus eyes open. The default mode network might be of particular interest, as during creative cognition activity in the default mode network is likely to reflect the spontaneous generation of ideas (Beaty et al., 2016).

### Practical Applications

The present study does not only enhance our scientific understanding of creativity, but may also provide important practical implications. Creative thinking can be considered one of the key competencies for the 21 century, and during the last years several means to enhance creativity have been developed. However, many of them are cost and time intensive. Eye-closure is a promising means to enhance creative thinking as it can be applied in various educational and organizational settings when creative thinking is needed. For example, educators can instruct students to close their eyes when thinking of creative ideas for an essay, and practitioners can organize more effective brainstorming sessions by identifying situations where the creative thinking process can be enhanced by eye-closure.

## Conclusion

Neuroscientific research has associated creativity with alpha oscillations, and alpha oscillations are hypothesized to protect internal mental processes from distracting external sensory information. The current study tested the hypothesis that excluding external information through eye-closure will benefit divergent and convergent creativity. The current findings provide a novel and easily applicable means to enhance divergent and convergent creativity through eye-closure.

## Author Contributions

SR, JA, and HvS developed the design of the study and wrote the manuscript. JA collected the data. JA and HvS analyzed the data.

## Conflict of Interest Statement

The authors declare that the research was conducted in the absence of any commercial or financial relationships that could be construed as a potential conflict of interest.

## Supplementary Material

TABLE S1 Words and promts used in the divergent and convergent thinking tasks.
