# Supplementary material for: Eye-Closure Enhances Creative Performance on Divergent and Convergent Creativity Tasks
Source: Front Psychol. 2018 Jul 31;9:1315. doi: 10.3389/fpsyg.2018.01315 (PMC6079281; doi:10.3389/fpsyg.2018.01315)

**DIVERGENT THINKING**

**Adapted Alternative Uses (AAU) test**

| Sieb |
| --- |
| Gartenschere |
| Streichholz |
| Gummiband |
| Schwamm |
| Kleiderhaken |
| Kerze |
| Scheckkarte |
| Handschuh |
| Zahnbürste |
| Buch |
| Tasse |
| Bügeleisen |
| Nussknacker |
| Schwimmflügel |
| Pflaster |
| Strohhalm |
| Messer |
| Weinglas |
| Ehering |
| Autoreifen |
| Bilderrahmen |
| Bettdecke |
| Gartenerde |
| Flasche |
| Haarbürste |
| Pinzette |
| Gürtel |
| Mixer |
| Pinsel |
| Büroklammer |

**Sentence Construction (SC) test**

| DRPS |
| --- |
| EABD |
| PIGD |
| ARHO |
| GMLI |
| IPBZ |
| BAHS |
| ZOWD |
| RAHV |
| PRGN |
| TKSN |
| LKID |
| BRSM |
| GSNH |
| IGBF |
| BTAG |
| NHRP |
| EMRS |
| LUIG |
| GRPA |
| BAEK |
| GFBH |
| GSNH |
| PKES |
| JMTL |
| EMDK |
| RPDS |
| TISG |
| DHIK |
| BFAD |

**CONVERGENT THINKING**

**Remote Associate Test (RAT)**

| **Item** | **Solution** |
| --- | --- |
| Schiff /  Bügeleisen /  Lock | Dampf |
| Herd / Heizung / Maske | Gas |
| Flocke / Sturm / Ball | Schnee |
| Fisch / Gelb / Mine | Gold |
| Bett / See / Herz | Krank |
| Kuh / gelb /Kuchen | Käse |
| Spiel / Schein / Bank | Geld |
| Lücke / Fee / Gold | Zahn |
| Flieger / Zeitung / Geld | Papier |
| Feder/ Klavier / Gebäude | Flügel |
| Daumen / Tee / Broccoli | Grün |
| Schnee / Korb / Tennis | Ball |
| Büro / Holz / Sitzen | Stuhl |
| Geburtstag/ Licht / Laterne | Kerze |
| Nacht / Tief / Störung | Schlaf |
| Bohne/ Pause / Schwarz | Kaffee |
| Hochzeit/ Zucker / Backen | Kuchen |
| Papier/ Basketball/Waesche | Korb |
| Spinne/Fischen/Tennis | Netz |
| Herz / Walzer / Trommel | Rhythmus |
| Baum / Gelb / Sauer | Zitrone |
| Dreieck / Wueste / Stein | Pyramide |
| Atem/Flugzeug/Ballon | Luft |
| Farben/Nagel/Hut | Finger |
| Ehe/Rund/Boxen | Ring |
| Familie/Apfel/Blätter | Baum |
| Tennis/Sitzen/Holz | Tisch |
| Lakritz/Langsam/Haus | Schnecke |
| Kabel/Vorwärts/Schrift | Rolle |
| Wein/Fenster/Tisch | Glas |
| Panzer/Langsam/Reptil | Schnecke |

**Word Construction (WC) test**


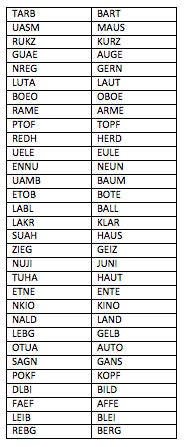

Supplement: TABLE S1 — Words and promts used in the divergent and convergent thinking tasks. [file Table_1.DOCX]
